# Effects of Baseline CSF α-Synuclein on Regional Brain Atrophy Rates in Healthy Elders, Mild Cognitive Impairment and Alzheimer’s Disease

**DOI:** 10.1371/journal.pone.0085443

**Published:** 2013-12-31

**Authors:** Niklas Mattsson, Philip Insel, Duygu Tosun, Jing Zhang, Clifford R. Jack Jr, Douglas Galasko, Michael Weiner, for the Alzheimer’s Disease Neuroimaging Initiative

**Affiliations:** 1 Department of Veterans Affairs Medical Center, Center for Imaging of Neurodegenerative Diseases, San Francisco, California, United States of America; 2 Clinical Neurochemistry Laboratory, Institute of Neuroscience and Physiology, the Sahlgrenska Academy at the University of Gothenburg, Mölndal, Sweden; 3 Department of Radiology and Biomedical Imaging, University of California, San Francisco, California, United States of America; 4 Department of Pathology, University of Washington, Seattle, WA, United States of America; 5 Department of Radiology, Mayo Clinic, Rochester, Minnesota, United States of America; 6 Department of Neurosciences, University of California, San Diego, California, United States of America; University of Antwerp, Belgium

## Abstract

**Background:**

Cerebrospinal fluid (CSF) α-synuclein is reduced in synucleinopathies, including dementia with Lewy bodies, and some studies have found increased CSF α-synuclein in Alzheimer’s disease (AD). No study has explored effects of CSF α-synuclein on brain atrophy. Here we tested if baseline CSF α-synuclein affects brain atrophy rates and if these effects vary across brain regions, and across the cognitive spectrum from healthy elders (NL), to patients with mild cognitive impairment (MCI) and AD.

**Methods:**

Baseline CSF α-synuclein measurements and longitudinal structural brain magnetic resonance imaging was performed in 74 NL, 118 MCI patients and 55 AD patients. Effects of baseline CSF α-synuclein on regional atrophy rates were tested in 1) four pre-hoc defined regions possibly associated with Lewy body and/or AD pathology (amygdala, caudate, hippocampus, brainstem), and 2) all available regions of interest. Differences across diagnoses were tested by assessing the interaction of CSF α-synuclein and diagnosis (testing NL versus MCI, and NL versus AD).

**Results:**

The effects of CSF α-synuclein on longitudinal atrophy rates were not significant after correction for multiple comparisons. There were tendencies for effects in AD in caudate (higher atrophy rates in subjects with higher CSF α-synuclein, P=0.046) and brainstem (higher atrophy rates in subjects with lower CSF α-synuclein, P=0.063). CSF α-synuclein had significantly different effects on atrophy rates in NL and AD in brainstem (P=0.037) and caudate (P=0.006).

**Discussion:** With the possible exception of caudate and brainstem, the overall weak effects of CSF α-synuclein on atrophy rates in NL, MCI and AD argues against CSF α-synuclein as a biomarker related to longitudinal brain atrophy in these diagnostic groups. Any effects of CSF α-synuclein may be attenuated by possible simultaneous occurrence of AD-related neuronal injury and concomitant Lewy body pathology, which may elevate and reduce CSF α-synuclein levels, respectively.

## Introduction

Cerebrospinal fluid (CSF) α-synuclein is reduced in most patients with synucleinopathies, including Parkinson’s disease (PD) and dementia with Lewy bodies (DLB) [1–4]. This may be due to entrapment of α-synuclein in Lewy bodies, similarly to how reduction of CSF β-amyloid42 (Aβ42) in Alzheimer’s disease (AD) may be caused by deposition of Aβ42 in senile plaques [5]. About 40-50 % of AD patients have α-synuclein positive Lewy bodies [6–8]. Studies of CSF α-synuclein levels in AD have shown mixed results, which may be due to methodological differences in sample handling and study cohorts [1,2,9–12]. Recent reports from the Alzheimer’s Disease Neuroimaging Initiative (ADNI) found increased CSF α-synuclein in patients with mild cognitive impairment (MCI) and AD [13], and demonstrated correlations between CSF levels of α-synuclein and the neuronal damage marker tau, across several diagnostic groups [14]. Such correlations suggest that CSF α-synuclein may be released in response to neuronal injury. Taken together, the data on CSF α-synuclein in AD and synucleinopathies suggest that two opposite forces may act on CSF α-synuclein levels in patients with neurodegeneration, with Lewy body pathology leading to lower CSF levels, and AD-type neuronal injury leading to higher CSF levels of the protein.

AD patients with Lewy body pathology have accelerated cognitive decline [15], suggesting that concomitant Lewy body pathology increases neuronal damage in AD. However, patients with DLB have a pattern of brain atrophy that is different from AD patients, with more engagement of midbrain structures, and less hippocampal and temporoparietal atrophy [16]. Given the associations between CSF α-synuclein and DLB, it is possible that CSF α-synuclein levels may influence brain atrophy patterns. In this study, our goal was to explore if CSF α-synuclein is related to brain atrophy rates in healthy elders (NL), patients with MCI and patients with AD. Structural magnetic resonance imaging (MRI) has been used to study effects of other CSF biomarkers on brain atrophy, including Aβ42 and tau [17,18], but to our knowledge no study has explored effects of CSF α-synuclein on brain atrophy.

We tested the a priori hypothesis that baseline levels of CSF α-synuclein have effects on regional atrophy rates. We tested this primarily in amygdala (which is a region where AD patients are likely to have both Lewy body pathology [6] and early atrophy [19]), brainstem (which is an early site of Lewy body accumulation in PD and DLB [20]), caudate (which is one of the subcortical structures affected in DLB[21,22]), and hippocampus (which is typically affected by atrophy in AD, but spared in DLB[23] ). Secondarily we tested effects of CSF α-synuclein on atrophy rates in all available grey matter and ventricle regions. We also tested the hypothesis that the effects of baseline CSF α-synuclein on brain atrophy rates vary across the cognitive spectrum from NL, to MCI and AD, by examining interactions between diagnoses and CSF α-synuclein.

## Methods

Data used in the preparation of this article were obtained from the ADNI database (adni.loni.ucla.edu). Details about the ADNI are given in the Acknowledgments section. Written informed consent was obtained from all participants and the study was conducted with prior institutional ethics approval.

### Study design

We examined volumetric changes in regions of interest (ROI) in NL, MCI and AD. Structural magnetic resonance imaging brain scans at multiple time points (up to 6 time points: ADNI screening, 6 months, 12 months, 18 months, 24 months, 36 months and 48 months, see Table 1) were acquired at multiple ADNI sites using 1.5 Tesla MRI scanners. Using FreeSurfer longitudinal processing framework, regional gray matter volume throughout the entire cortex was automatically measured at each time point. Linear mixed effect models were performed to test if baseline CSF α-synuclein was associated with rates of change in volume.

**Table 1:** Demographics.

	**NL**	**MCI**	**AD**
**N, baseline**	74	118	55
**N follow-up at 6, 12, 18, 24, 36, 48 months**	71, 67, 0, 61, 47, 12	112, 105, 91, 82, 54, 13	53, 45, 0, 33, 0, 0
**Sex, F/M**	36/38	40/78	23/32
**Baseline CSF α-synuclein (ng/mL), mean (SD)**	0.42 (0.14)	0.49 (0.18)	0.54 (0.18)
**APOE ε4 carriers, +/-**	17/57	66/52	35/20
**Education (years), mean (SD)**	15.9 (2.5)	15.6 (3.0)	15.3 (3.2)
**Baseline MMSE, mean (SD)**	29.1 (1.1)	26.8 (1.9)	23.6 (1.9)
On AD drug at baseline* (%)	0 (0%)	65 (55%)	51 (93%)

^*^ AD drugs were galantamine, donepezil, rivastigmine or memantine. Subjects were considered using an AD drug at baseline if this was indicated in the available data records for a time-point +/- 6 months from the lumbar puncture.

### Participants

This was an ADNI study. The ADNI was launched in 2003 by the National Institute on Aging (NIA), the National Institute of Biomedical Imaging and Bioengineering (NIBIB), the Food and Drug Administration (FDA), private pharmaceutical companies and non-profit organizations, as a $60 million, 5-year public-private partnership. The primary goal of ADNI has been to test whether serial MRI, PET, other biological markers, and clinical and neuropsychological assessment can be combined to measure the progression of MCI and early AD. Determination of sensitive and specific markers of very early AD progression is intended to aid researchers and clinicians to develop new treatments and monitor their effectiveness, as well as lessen the time and cost of clinical trials. The Principal Investigator of this initiative is Michael W. Weiner, MD, VA Medical Center and University of California – San Francisco. ADNI is the result of efforts of many co-investigators from a broad range of academic institutions and private corporations, and subjects have been recruited from over 50 sites across the U.S. and Canada. The initial goal of ADNI was to recruit 800 subjects but ADNI has been followed by ADNI-GO and ADNI-2. To date these three protocols have recruited over 1500 adults, ages 55 to 90, to participate in the research, consisting of cognitively normal older individuals (NL), people with MCI, and people with early AD. Inclusion criteria for NL subjects were mini-mental state examination (MMSE) scores 24-30, Clinical Dementia Rating (CDR) 0, no depression, no MCI, and no dementia. MCI subjects had MMSE 24-30, memory complaint, and objective memory loss (adjusted for education) measured by Wechsler Memory Scale Logical Memory II, CDR 0.5, absence of significant levels of impairment in other cognitive domains, essentially preserved activities of daily living, and no dementia. AD subjects had MMSE 20-26, CDR 0.5-1, and met the NINCDS-ADRDA criteria for probable AD. For up-to-date information on ADNI, see http://www.adni-info.org. The population in this study included ADNI-1 NL, MCI and AD subjects with valid results for CSF α-synuclein and successful FreeSurfer processing of MR images. The groups did not have matched numbers (74 NL, 118 MCI, 55 AD) because we made use of data from as many subjects as possible with both imaging and CSF.

### Structural MRI acquisition

The participants underwent a standardized 1.5 Tesla MRI protocol (http://www.loni.ucs.edu/ADNI/Research/Cores/index.shtml), which included two T1-weighted MRI scans using a sagittal volumetric magnetization prepared rapid gradient echo (MP-RAGE) sequence. Only one of the MP-RAGE sets was used for analysis. The ADNI MRI quality control center at the Mayo Clinic selected the MP-RAGE image with higher quality and corrected for system-specific image artifacts, as previously described [24].

### FreeSurfer longitudinal MR image processing

Automated cortical and subcortical volume measures were performed with FreeSurfer software package, version 4.4 (http://surfer.nmr.mgh.harvard.edu/fswiki) [25,26]. To reduce the confounding effect of intra-subject morphological variability, each subject’s longitudinal data series was processed by FreeSurfer longitudinal workflow. For a full description of the FreeSurfer processing steps, see 25,26, and for a full description of the longitudinal workflow, see http://surfer.nmr.mgh.harvard.edu/fswiki/LongitudinalProcessing. All images underwent standardized quality control procedures. Subjects with complete segmentation failure or gross errors throughout all brain regions were rated as complete failure and the ones with gross errors in one or more specific brain regions (i.e., temporal lobe regions, superior regions, occipital regions, and insula) were given partial pass rating. All the subjects with passing quality control rating were included in analyses presented in this work. Details of our quality control procedures are posted online at http://www.loni.ucla.edu/twiki/pub/ADNI/ADNIPostProc/UCS-FFreeSurferMethodsSummary.pdf.

For this study, we used volumetric measurements combining data from left and right hemispheres. We included data from all available grey matter and ventricle ROI (N=34 cortical ROI, N=9 subcortical ROI, N=5 ventricle ROI).

### CSF biomarker concentrations

A CSF sample from the lower spine of each subject was collected at baseline by lumbar puncture, as described in the ADNI procedures manual (http://www.adni-info.org). In brief, CSF was collected into collection tubes provided to each site, then transferred into polypropylene transfer tubes, and shipped frozen to the ADNI Biomarker Core laboratory at the University of Pennsylvania Medical Center for long term storage in -80° C. CSF α-synuclein was measured using a xMAP bead-based assay, as recently described [13,14]. Only samples with CSF hemoglobin < 200 ng/mL were used, to minimize the risk of contaminating CSF with blood-based α-synuclein [13].

### Statistical analyses

CSF α-synuclein data were logarithmized. Potentially confounding effects by age, sex, and *APOE* genotype on CSF α-synuclein were evaluated by non-parametric tests (Mann-Whitney test and Spearman Correlation tests, as appropriate). Several subjects lacked MRI data for one or more visits. A generalized linear mixed model was used to test if baseline CSF α-synuclein was associated with a binary missing indicator for each visit.

Linear mixed effects models were used to test if atrophy rates were significantly different from zero, with volume as the dependent variable, and time (in years) from the first image measurement as an independent fixed effect. To examine effects of CSF α-synuclein on regional rates of volume change, we tested the interaction CSF α-synuclein:time. To examine if the effect of CSF α-synuclein varied across diagnostic groups, we tested the interaction CSF α-synuclein:diagnosis:time, comparing NL versus AD, and NL versus MCI. All models were co-varied for all main sub-effects, *APOE* genotype, age and sex. All models included a random intercept, and most models also included a random slope (in a few regions, the models would not converge with a random slope, and these models were only tested with fixed effects and a random intercept).

After inspection of the correlation within subjects, a compound symmetry structure was assumed for the linear mixed effects models. We assessed the applicability of the models by evaluating linearity of regional volumes over time within subjects, and the normality of the model residuals. There were no significant outliers in the models of the a priori defined regions (in terms of model residuals). All available data points were included.

Data on volume and proteins in text, tables and figures, are standardized (centered on the mean and divided by the standard deviation).

All tests were two-sided and considered significant at P<0.05. When indicated in the manuscript, P-values were corrected for multiple comparisons, using a false discovery rate (fdr) correction. Corrections were applied to all tested regions in each diagnostic group (k=4 [*a priori* defined ROI] or k=48 [all available ROI]). All statistics were done using R (v. 2.15.2, The R Foundation for Statistical Computing). The software “Medical Image Processing, Analysis and Visualization” (MIPAV, v. 5.4.4, Center for Information Technology, National Institute of Health) was used to plot statistics on brain images.

## Results

The study included 74 NL, 118 MCI and 55 AD dementia patients. See table 1 for demographic data. During the follow-up of ADNI, 13 subjects converted from NL to MCI, 2 from NL to AD, 60 from MCI to AD, 3 from MCI to NL, and 1 from AD to MCI. In this study, all subjects were analyzed according to their diagnosis at study enrollment. Patients with MCI or AD were allowed symptomatic treatment for memory problems at the discretion of their treating physicians. Sixty-five of the MCI and 51 of the AD subjects were using AD-medications (galantamine, donepezil, rivastigmine or memantine) at the time of lumbar puncture (Table 1).

### Potential confounding factors

Age, sex, and *APOE* genotype were potential confounders of the relationship between baseline CSF α-synuclein and regional atrophy rates. We tested for imbalances between CSF α-synuclein and these factors, using non-parametric tests. This resulted in P=0.036 for *APOE* genotype (higher CSF α-synuclein in *APOE ε4* positive subjects), P=0.49 for age, and P=0.65 for sex. In the results presented in this paper on the effects of CSF α-synuclein on atrophy rates, we adjusted all models for *APOE* genotype, age and sex. However, the main results were only marginally different when not adjusting for these co-variates (data not shown).

### Missing data

Several subjects lacked MRI data for one or more visit (see Table 1), but the *CSF* α-synuclein:time interaction effect was not significantly associated with missing visits (P=0.13), suggesting that study drop-out was not associated with baseline CSF α-synuclein.

### Atrophy rates in a priori defined regions

**Figure 1:**
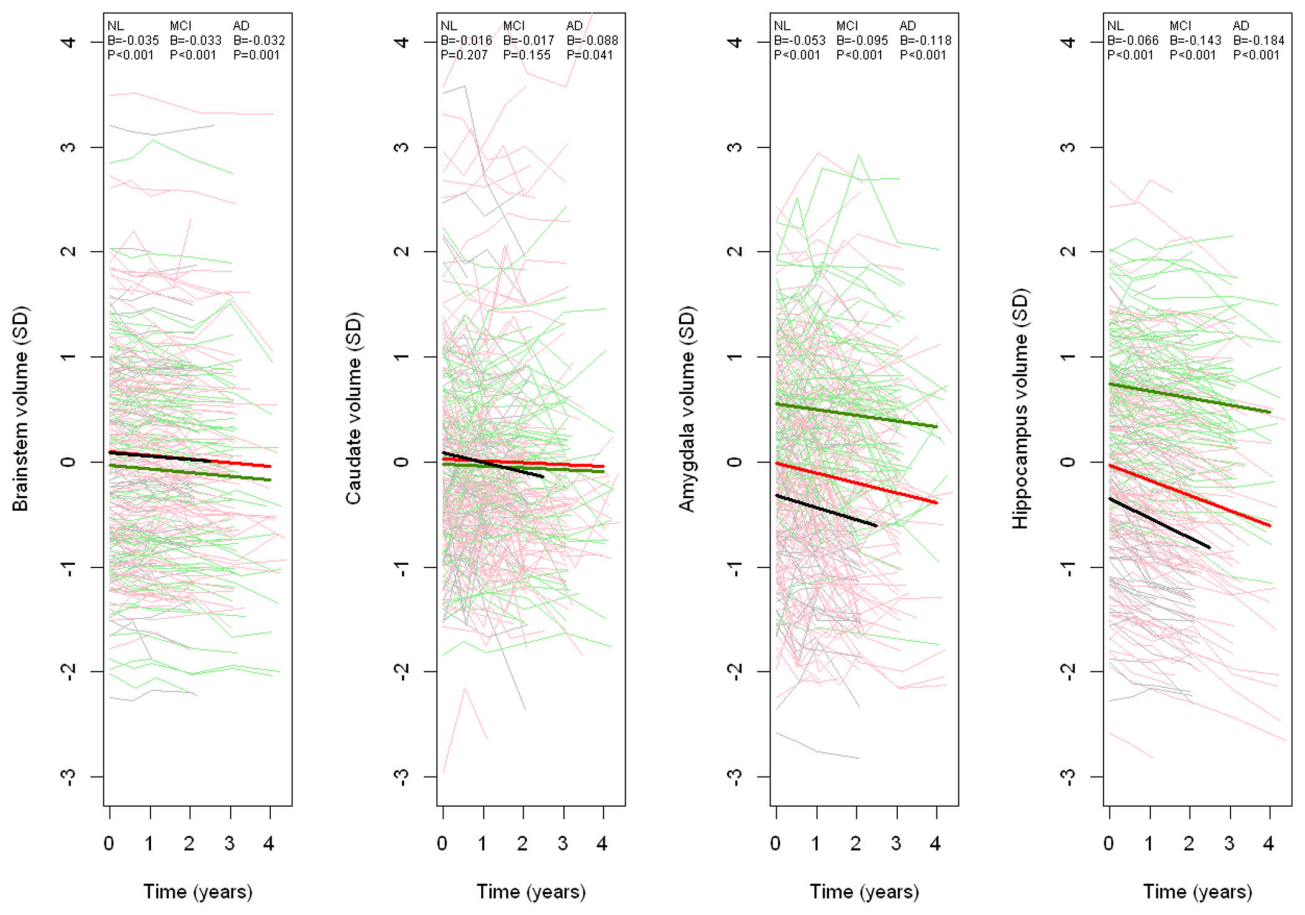
Longitudinal volume changes in a priori defined regions. Y-axes show volumes and x-axes show time from study onset. The thin lines are observed volumes (standardized) in NL (green), MCI (red), and AD (black). The thick lines are fitted using linear mixed effects models with time as fixed effects, adjusted for age, APOE and sex, modeled within each diagnostic group. B-coefficients and p-values are presented for the time effect.

The *a priori* defined primary ROI for this study were brainstem, caudate, amygdala and hippocampus. See figure 1 for plots of observed volumes over time in NL, MCI and AD, including fitted lines for linear mixed models adjusted for age, *APOE* and sex. The patterns of atrophy rates differed across the subgroups. In brainstem, all groups had significant and similar atrophy rates (P≤0.001, β=-0.032 to -0.035, standardized volumes). In caudate, the atrophy rate was significantly different from zero only in AD (P=0.041, β=-0.088). In amygdala and hippocampus, all groups had significant atrophy rates, with steeper slopes in AD (amygdala, P<0.001, β=-0.118; hippocampus, P<0.001, β=-0.184) and MCI (amygdala, P<0.001, β=-0.095; hippocampus, P<0.001, β=-0.143) than in NL (amygdala, P<0.001, β=-0.053; hippocampus, P<0.001, β=-0.066). We tested the interactions between diagnoses and time to assess if the differences in slopes were significant, and found significantly different slopes for AD versus NL in caudate (P=0.021), MCI versus NL in amygdala (P=0.022), AD versus NL in amygdala (P=0.023), MCI versus NL in hippocampus (P<0.001), and AD versus NL in hippocampus (P<0.001). The p-values in this paragraph are not adjusted for multiple comparisons, since they are not part of the primary aim of the study.

### Effects of CSF α-synuclein on atrophy rates in a priori defined regions

**Figure 2:**
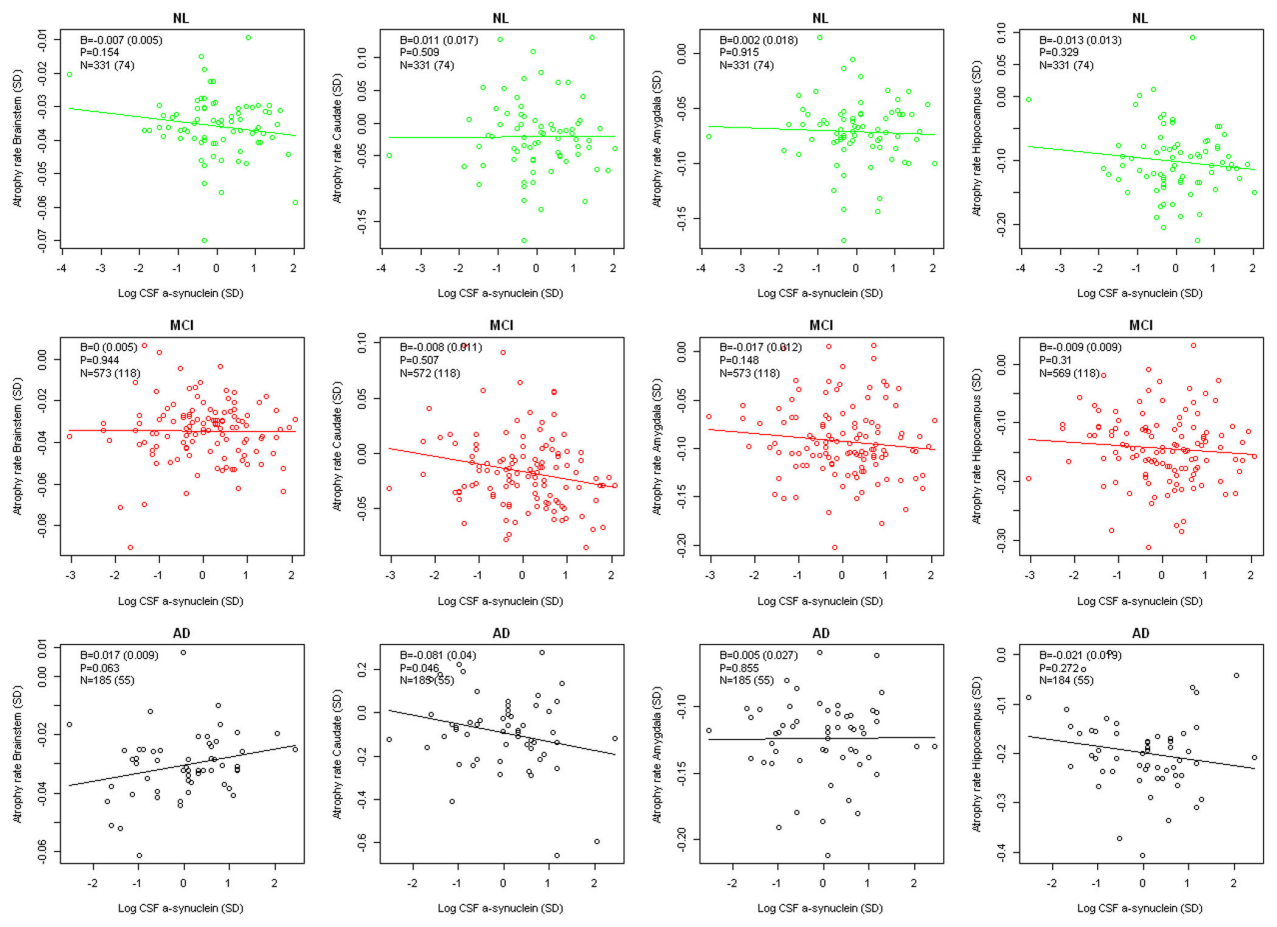
Longitudinal volume changes in relation to baseline CSF α-synuclein in *a priori* defined regions. Data for the *a* *priori* defined regions of interest in NL (top row), MCI (middle row), and AD (bottom row). Y-axes show atrophy rates in individual subjects, calculated by linear mixed effects models, adjusted for age, APOE and sex. X-axes show baseline CSF α-synuclein levels. Regression lines for CSF α-synuclein linearly regressed on adjusted atrophy rates. In each panel, β-coefficients (standard errors in brackets), p-values (not corrected for multiple comparisons), and number of observations (number of subjects in brackets) are from the interaction CSF α-synuclein:time in the linear mixed effects model: volume ~ CSF α-synuclein:time + age + APOE + sex. No p-values were significant after correction for multiple comparisons by a false discovery rate correction (correcting within study group [4 tests]).

We next tested if baseline CSF α-synuclein had effects on atrophy rates in the *a priori* defined ROI, within the groups of NL, MCI and AD patients. Effects were adjusted for age, *APOE* and sex. There were no significant effects of CSF α-synuclein after correction for multiple comparisons. See figure 2 for results. In AD, there were tendencies towards effects in caudate, with higher atrophy rates in subjects with high baseline CSF α-synuclein (uncorrected P=0.046), and in brainstem (uncorrected P=0.063), with higher atrophy rates in subjects with low baseline CSF α-synuclein. When modeling all subjects simultaneously, CSF α-synuclein had a significant effect on atrophy rate in hippocampus (β=-0.021, SE=0.0065, P=0.0011, not corrected for multiple comparisons, hippocampal volume and CSF α-synuclein centered and standardized in all subjects), but not in the other regions.

### Diagnosis dependent effects of CSF α-synuclein on atrophy rates in a priori defined regions

We next compared effects of baseline CSF α-synuclein on atrophy rates across diagnostic groups, testing NL versus MCI, and NL versus AD. For NL versus AD, significant CSF α-synuclein:diagnosis:time interactions were found in 1) the brainstem, where AD patients with low baseline CSF α-synuclein had higher atrophy rates while the opposite was seen in NL (interaction effect, β=0.026, SE=0.011, P=0.037), and 2) the caudate, where AD patients with high baseline CSF α-synuclein had higher atrophy rates while the opposite was seen in NL (interaction effect, β=-0.125, SE=0.039, P=0.006). The interaction in caudate was not present after removal of two AD subjects with high CSF α-synuclein and large atrophy rates.

### Effects of CSF α-synuclein on volume changes in all regions

**Figure 3:**
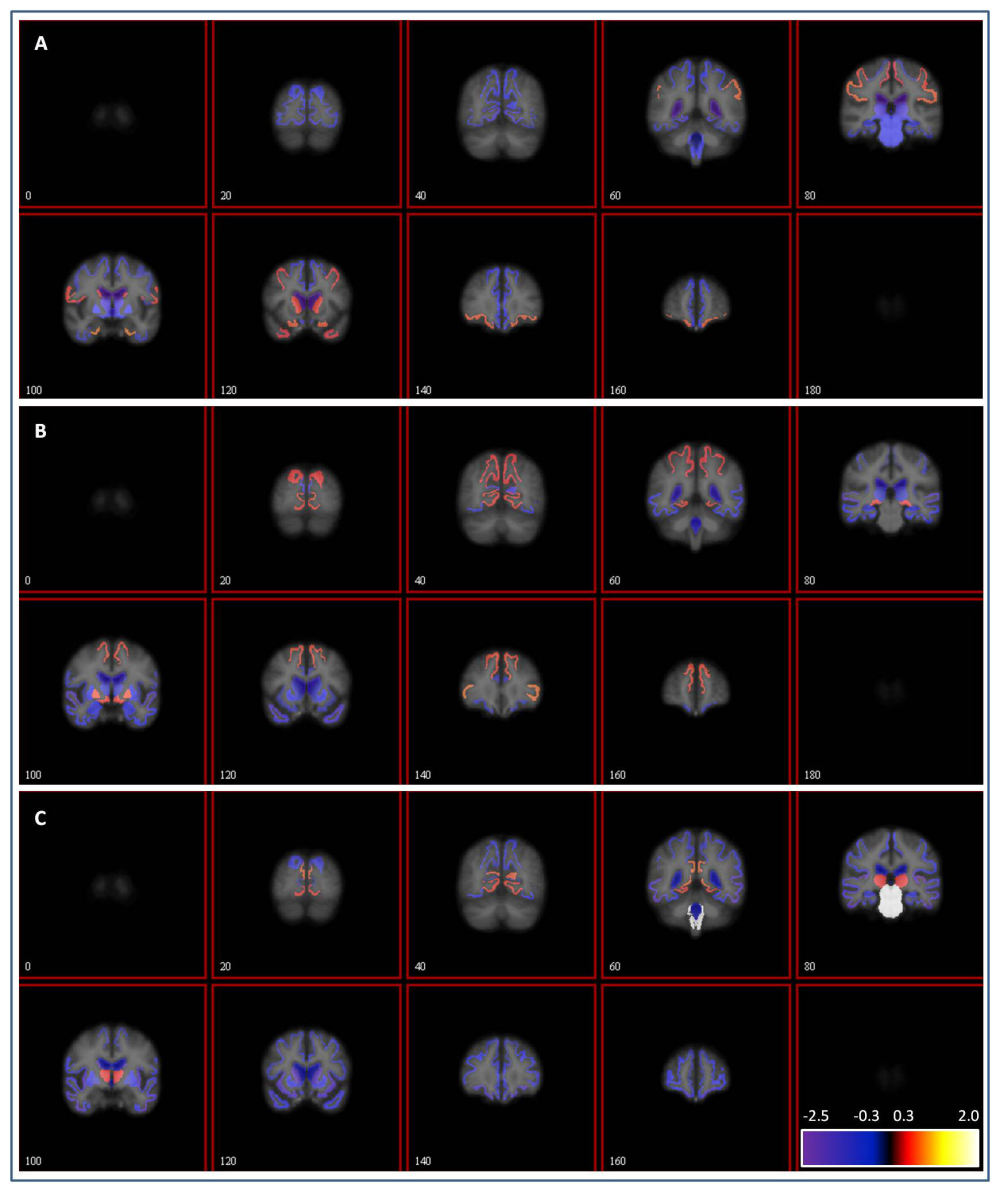
Exploratory analysis of longitudinal volume changes in relation to baseline CSF α-synuclein. Results from an exploratory analysis of effects of CSF α-synuclein on volume change in all available regions. Statistics for FreeSurfer regions were plotted on a T1 brain image template. Colors indicate regional t-values for the interaction CSF α-synuclein:time in linear mixed effects models, adjusted for APOE, sex and age, in NL (panel A), MCI (panel B), and AD (panel C). Increasingly positive t-values are indicated by red-yellow-white colors and increasingly negative t-values by blue-purple colors. Regions with T-values > -0.30 and < 0.30 (roughly corresponding to P-values > 0.75) are not colored (see scale). Note that the sign of the t-value depends on the relationship between CSF α-synuclein and volume change. Thus, in regions with negative volume change (atrophy), a negative t-value indicates that higher atrophy rates were seen in subjects with high CSF α-synuclein, and a positive t-value that higher atrophy rates were seen in subjects with low CSF α-synuclein. In contrast, in the few regions where the longitudinal volume change was positive (ventricles and pallidum in NL, MCI and AD, and pericalcarine in MCI), a negative t-value indicates that volume enlargement was seen in subjects with low CSF α-synuclein, and a positive t-value that volume enlargement was seen in subjects with high CSF α-synuclein. No effect was significant when correcting for multiple comparisons (correcting within study group [48 tests]).

We finally tested effects of CSF α-synuclein on longitudinal volume changes in all available ROI (N=48) in NL, MCI and AD tested separately. No longitudinal change was significantly affected by baseline CSF α-synuclein after fdr correction for multiple comparisons. Before correcting for multiple comparisons, there were effects in NL in the lateral ventricle (β=-0.018, SE=0.007, P=0.011); and in AD in the transverse temporal region (β=-0.068, SE=0.029, P=0.020), in the banks of the superior temporal sulcus (β=-0.047, SE=0.024, P=0.046), in the caudate (β=-0.081, SE=0.040, P=0.046), and in the inferior temporal region (β=-0.033, SE=0.017, P=0.047). To visualize this exploratory analysis, we generated whole brain maps showing t-values for the effects of CSF α-synuclein on regional volume change (Figure 3, panels A-C). In NL and MCI, atrophy rates in some regions tended to be associated with low baseline CSF α-synuclein levels (including the supramarginal and entorhinal regions in NL, Figure 3, panel A; and pars triangularis and superior frontal in MCI, Figure 3, panel B), while atrophy rates in other regions tended to be associated with high baseline CSF α-synuclein levels (including the thalamus in NL, Figure 3, panel A, and superior temporal and the temporal pole in MCI, Figure 3, panel B). In AD, atrophy rates were most commonly associated with high baseline CSF α-synuclein levels, with the brainstem being the most prominent exception, where atrophy rates were associated with low baseline CSF α-synuclein (Figure 3, panels C). We noted that the largest longitudinal increases of ventricle volumes were generally seen in subjects with low baseline CSF α-synuclein (scatter-plot data available in Figure S1), while the largest atrophy rates in grey matter regions generally were seen in subjects with high baseline CSF α-synuclein. 

## Discussion

The key findings of this study, which to our knowledge is the first study on effects of CSF α-synuclein on brain atrophy, were that (1) baseline CSF α-synuclein had no significant effects on atrophy rates in amygdala, caudate, brainstem and hippocampus in the individual groups of NL, MCI and AD; (2) when comparing groups, there were some significant differences across the diagnostic groups, with stronger effects of CSF α-synuclein on atrophy rates in AD than in NL in caudate and brainstem; (3) in an exploratory analysis of all available brain regions, the effects of CSF α-synuclein seemed to vary across brain regions and diagnosis, although no effects reached statistical significance after correcting for multiple comparisons.

The first major finding of this study was that baseline CSF α-synuclein lacked significant effects on atrophy rates in the *a priori* defined brain regions amygdala, caudate, brainstem and hippocampus, within the diagnostic groups. This finding argues against the use of CSF α-synuclein as a stand-alone indicator of future risk of brain atrophy within the cognitive spectra from NL to MCI and AD. It is possible that the combination of CSF α-synuclein with other biomarkers have stronger effects on brain atrophy than individual biomarkers. A recent study found that the combination of CSF α-synuclein with the core CSF AD biomarkers Aβ42, total tau (T-tau) and phosphorylated tau (P-tau) enhances the diagnostic accuracy for MCI to AD conversion compared to the individual biomarkers [14]. Future studies on CSF α-synuclein and brain imaging may build on this and incorporate CSF Aβ42, T-tau and P-tau, and for example test if low CSF α-synuclein increases the risk for DLB-type pathology in subjects where other biomarkers strongly indicate AD.

The second major finding of this study was that although the effects of CSF α-synuclein on atrophy rates were non-significant within the individual diagnostic groups, there were significant differences between NL and AD in brainstem and caudate, when comparing these diagnostic groups with each other. In the brainstem, AD patients with low baseline CSF α-synuclein had high atrophy rates, while the opposite was seen in NL. A possible interpretation of this is that concurrent Lewy body pathology, as reflected by low CSF α-synuclein levels, are more strongly associated with brainstem atrophy in AD than in NL. The brainstem is interesting, since it is a typical region for early Lewy body deposits [20]. However, it should be noted that the brainstem is not a typical region for AD-related brain atrophy, and the development of Lewy body pathology does not always begin in the brainstem [27]. Also, in this study the overall brain stem atrophy rates were similar between NL, MCI and AD. In the caudate, the relationship between CSF α-synuclein and atrophy rate was reversed, so that AD patients with high baseline CSF α-synuclein had high atrophy rates, while the opposite was seen in NL. We have no clear explanation for this finding. We noted that two outliers with high CSF α-synuclein levels and high atrophy rates affected the slope in the AD subjects. Therefore these results must be treated with caution. Further studies are needed to elucidate if CSF α-synuclein affects brain atrophy differently across NL, MCI and AD in regions implicated in DLB and AD.

The final part of this study was an exploratory analysis of all available regions. After correction for multiple comparisons, there were no significant effects of CSF α-synuclein. However, although the effects were non-significant, the patterns seemed to differ between NL and MCI/AD. In NL and MCI, the effects of CSF α-synuclein varied, and atrophy rates were related to either high or low CSF α-synuclein (although non-significantly). In AD, high atrophy rates tended to be related to high CSF α-synuclein, with some exceptions, for example the brainstem, where high atrophy rates tended to be related to low CSF α-synuclein. This difference between groups may be related to random variations, since atrophy rates were larger in AD. We observed that longitudinally increased ventricle volumes generally were related to low CSF α-synuclein. Further studies of white matter structure and lesions may be useful to clarify if atrophy of periventricular regions is related to Lewy body pathology, as reflected by low CSF α-synuclein.

It should be emphasized that this was not a study of subjects afflicted with disorders known to be primarily associated with α-synuclein pathology. Both PD and DLB are characterized by the presence of Lewy Bodies and α-synuclein in the brain. Patients with PD are being studied in the Parkinsons Progressive Biomarkers Initiative and CSF α-synuclein is being collected from these patients. Therefore, the results of the PPMI project (which are publically available on the UCLA LONI Website) will be interesting to compare with the ADNI data. We know of no study measuring longitudinal atrophy in DLB subjects with CSF α-synuclein measurements.

Limitations of this study include the small sample size, and the lack of autopsy follow-up. Also, since only a subset of AD and MCI patients are likely to have concomitant Lewy body pathology, it is possible that a larger study cohort is needed to detect any effects of CSF α-synuclein on Lewy body related atrophy. This is especially true if CSF α-synuclein is affected by opposite forces in AD, with Lewy body pathology reducing levels and neuronal injury increasing levels, attenuating any effects of the biomarker, as has recently been suggested [14]. Another limitation of this study is that we only considered effects on grey matter atrophy and ventricle volume. It is possible that CSF α-synuclein levels also affects white matter integrity, which may be tested in future studies using tractography.

## Conclusions

This is the first study of CSF α-synuclein and brain atrophy rates in NL, MCI and AD. The findings were generally negative, with small or no effects of baseline CSF α-synuclein and atrophy rates. Future studies may incorporate other biomarkers to identify AD patients with likely concomitant Lewy body pathology, and test if these patients have unique relationships between CSF α-synuclein and patterns of atrophy.

## Supporting Information

Figure S1 **Y-axes show slopes for individual subjects from linear mixed effects models, adjusted for age, and x-axes show baseline CSF α-synuclein levels.** The panels show data for the lateral ventricles in NL, MCI and AD. Regression lines were generated by regressing CSF α-synuclein levels on the slope. B-coefficients and p-values are presented for the interaction CSF α-synuclein:time.
(TIFF)
