# Supplementary figures and images for: Effects of Baseline CSF α-Synuclein on Regional Brain Atrophy Rates in Healthy Elders, Mild Cognitive Impairment and Alzheimer’s Disease
Source: PLoS One. 2013 Dec 31;8(12):e85443. doi: 10.1371/journal.pone.0085443 (PMC3877372; doi:10.1371/journal.pone.0085443)

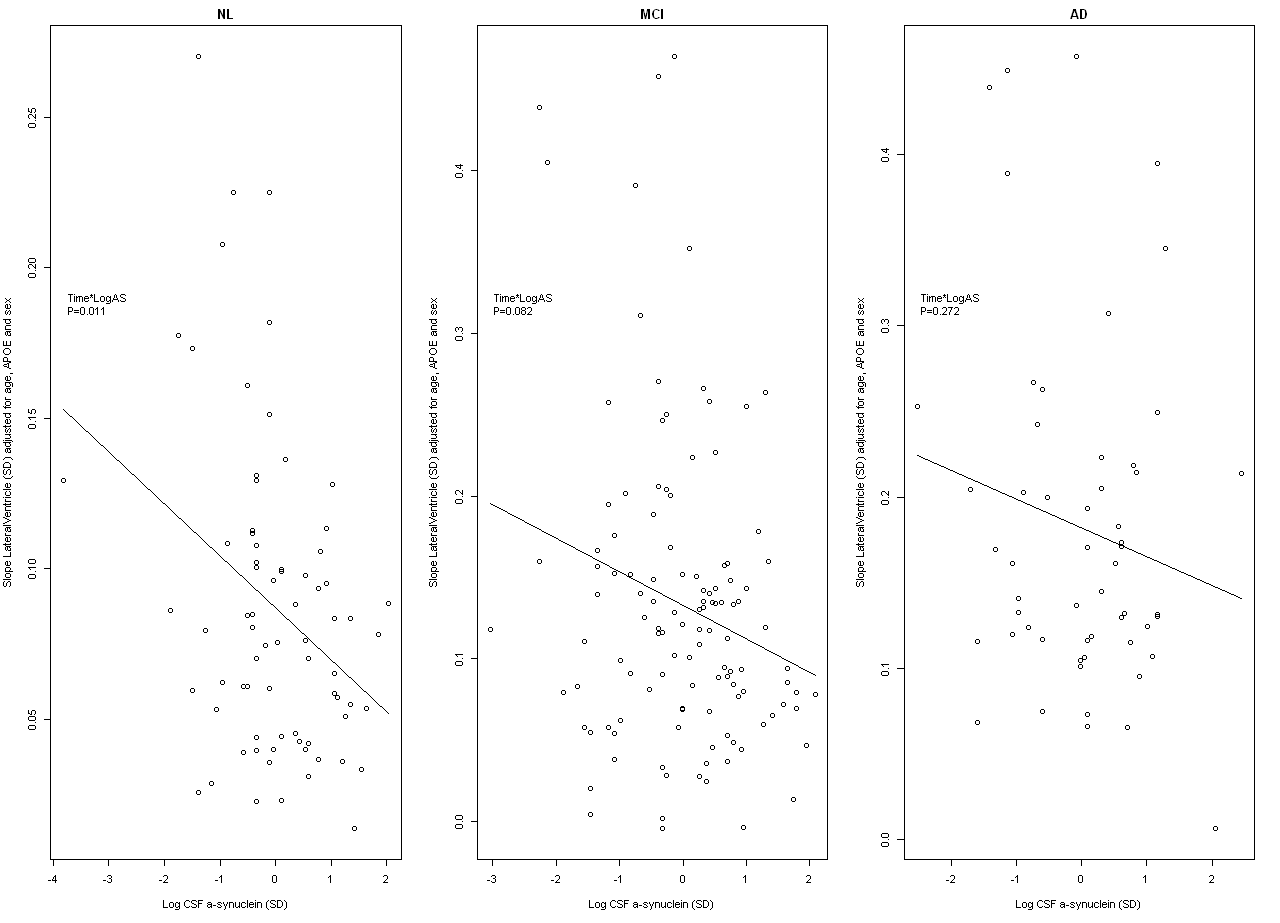

Supplement: Figure S1 — Y-axes show slopes for individual subjects from linear mixed effects models, adjusted for age, and x-axes show baseline CSF α-synuclein levels. The panels show data for the lateral ventricles in NL, MCI and AD. Regression lines were generated by regressing CSF α-synuclein levels on the slope. B-coefficients and p-values are presented for the interaction CSF α-synuclein:time. (TIFF) [file pone.0085443.s001.tiff]
